# β1 Adrenergic Receptor Polymorphisms and Heart Failure: A Meta-Analysis on Susceptibility, Response to β-Blocker Therapy and Prognosis

**DOI:** 10.1371/journal.pone.0037659

**Published:** 2012-07-17

**Authors:** Wen-Nan Liu, Kai-Li Fu, Hai-Yang Gao, Yuan-Yuan Shang, Zhi-Hao Wang, Gui-Hua Jiang, Yun Zhang, Wei Zhang, Ming Zhong

**Affiliations:** 1 Key Laboratory of Cardiovascular Remodeling and Function Research, Chinese Ministry of Education and Chinese Ministry of Public Health, Ji’nan, People’s Republic of China; 2 Department of Cardiology, Qilu Hospital of Shandong University, Ji’nan, People’s Republic of China; Temple University, United States of America

## Abstract

**Aims:**

The risk stratification of patients for heart failure (HF) remains a challenge, as well as the anticipation of the response to β-blocker therapy. Since the pivotal role of β1 adrenergic receptor (β1-AR) in HF, many publications have studied the associations between the β1-AR polymorphisms (Ser49Gly and Arg389Gly) and HF, with inconsistent results. Thus, we performed a meta-analysis of studies to evaluate the impact of β1-AR polymorphisms on susceptibility to HF, the response to β-blocker therapy and the prognosis of HF.

**Methods and Results:**

Electronic databases were systematically searched before August 2011. We extracted data sets and performed meta-analysis with standardized methods. A total of 27 studies met our inclusion criteria. It was found that in East Asians, the Gly389 allele and Gly389 homozygotes significantly increased the HF risk, while the Gly389 allele and Gly389 homozygotes trended to decrease the risk of HF in whites. With the similar reduction of heart rate, overall, the Arg389 homozygotes showed a better response to β-blocker therapy. Furthermore, the Arg389 homozygotes were significantly associated with better LVEF improvement in East Asians and a mixed population. And in white people, the Arg389 homozygotes made a greater LVESd/v improvement and trended to be associated with better LVEDd/v improvement. However, the prognosis of Arg389 homozygotes HF patients was similar to those with Gly389 carriers. The Ser49Gly polymorphism did not impact the risk or prognosis of HF.

**Conclusion:**

Based on our meta-analysis, the Gly389 allele and Gly389 homozygotes were risk factors in East Asians while trending to protect whites against HF. Furthermore, Arg389 homozygote is significantly associated with a favorable response to β-blocker treatment in HF patients. However, neither of the two polymorphisms is an independent predictor of the prognosis of HF.

## Introduction

Heart failure (HF) is the end-stage of various heart diseases, and it represents a major health problem owing to its high prevalence, morbidity, mortality and significant health-care costs [1]–[2]. β-blockers are mainstay of current treatment of heart failure (HF) in guideline, for their administration has beneficial effects on left ventricular (LV) function and prognosis [1]–[7]. However, in clinical practice, the response to β-blocker therapy and prognosis of HF are variable among patients. The purpose of diagnosing and treating HF is bringing about a reduction of mortality and morbidity. It is therefore important to identify the risk factors for HF so that preventive measurements can be undertaken early; and anticipation the response to β-blockers would help the physicians to make therapeutic decision. Thus there are needs for risk stratification tools of HF and predictors of response to β-blockers treatment, which are still challenges in the real world.

As the targets of endogenous catecholamines and β-blockers, β1 adrenergic receptor is known to play a pivotal role in the progression and treatment of HF [8]. Two common functional polymorphisms of the β1-AR, Ser49Gly and Arg389Gly have been considered as predictors of susceptibility to HF, response to β-blockers therapy and even prognosis of HF in some publications. However, being limited by small sample size, the results are controversial. Thus, we performed this meta-analysis of all the available studies to evaluate the impacts of β1 AR polymorphisms on susceptibility to HF, response to β-blocker therapy, and prognosis. It may provide information for further investigation on individualized HF prevention and treatment.

## Methods

### Search and Selection Process

Electronic searches by PubMed, Cochrane Library, and Chinese Biomedical Disc were used to identify published articles on β1 AR polymorphisms and heart failure. We combined search terms for “β1 receptor genetic polymorphism”, “ADRB1 polymorphism”, “Ser49Gly”, “Arg389Gly”, and all of the studies were published before August 2011. When more than one of the same patient population was included in several publications, only the most recent or complete study was identified in this meta-analysis.

### Inclusion Criteria

For the susceptibility to heart failure: (1) case–control studies, (2) evaluating the association between the β1 adrenergic receptor genetic polymorphisms and HF risk, (3) the diagnosis of HF was made according to the World Health Organization criteria, based on the presence of the typical clinical signs and symptoms of HF with left ventricular dysfunction.

Response to β-blockers therapy: (1) The definition of HF was based on the World Health Organization criteria, (2) evaluation of β1 AR genetic polymorphisms on the reduction of heart rate (ΔHR), the changes of left ventricular ejection fraction (ΔLVEF), left ventricular end-diastolic diameter/volume (ΔLVEDd/v), left ventricular end-systolic diameter/volume (ΔLVESd/v), with at least 3 months follow up, (3) all the patients received β-blockers therapy, other medicines such as ACEI/ARB, diuretics, spironolactone, and digoxin were used when necessary.

β1 AR polymorphisms and HF prognosis: (1) The definition of HF was based on the World Health Organization criteria, (2) evaluation of β1 adrenergic receptor genetic polymorphisms on all-cause mortality or combined end-point including death, heart transplantation and hospitalization, (3) patients were followed up for more than 1 year.

All the searches were restricted to articles in English or Chinese. Case reports, editorials, and review articles were excluded.

### Data Extraction

Two reviewers independently extracted data from published sources. The following information was extracted from each study: the first author, publication year, study design, ethnicity, sample size, distribution of genotypes, Hardy -Weinberg equilibrium (HWE) in controls, the parameters of therapy response (ΔHR, ΔLVEF, ΔLVEDd/v, and ΔLVESd/v), and the occurrence of death and combined end-point. We did not define any minimum number of subjects as required to include a study in our meta-analysis. If necessary data could not be extracted, the study authors were contacted by e-mail, with a reminder after 30 days. Disagreements were resolved by joint review and consensus.

### Statistics

Cochrane collaboration meta-analysis review methodology was used for this study [9]. The allele contrast, the recessive and dominant models were evaluated for association between the risk of heart failure and the β1 AR polymorphisms. In addition to the overall analysis, subgroup analysis for each ethnicity was also performed. Ethnicity was categorized into 3 main groups: (1) white descents, (2) East Asian descents, and (3) black descents. The distribution of the genotypes in the control group was tested for Hardy–Weinberg equilibrium [9], a *P*<0.05 was considered that the distribution of genotypes in the control group deviated from HWE. Studies with controls not in HWE were subjected to a sensitivity analysis.

In the meta-analysis of influence on HF patients therapy response and prognosis, comparisons of therapy response parameters (ΔHR, ΔLVEF, ΔLVEDd/v, ΔLVESd/v) and prognosis (mortality and combined end-point) between Gly49 carriers (Gly49Gly + Ser49Gly) and Ser49 homozygotes, Gly389 carriers (Gly389Gly + Arg389Gly) and Arg389 homozygotes were carried out respectively.

The analysis was carried out using Review Manager statistical software (RevMan version 5.0.2; The Nordic Cochrane Center, Regshospitalet). Pooled relative risk (RR) and associated 95% confidence intervals (CIs) were calculated for the risk and prognosis of HF. The therapy response was evaluated with weighted mean difference (WMD) for ΔHR and ΔLVEF, or standardized mean difference (SMD) for ΔLVEDd/LVEDv, ΔLVESd/LVESv. All tests and CIs were 2-sided, and a *P*<0.05 was considered statistically significant.

The presence of heterogeneity across studies was evaluated. The fixed-model (Mantel–Haenszel) was used when smaller heterogeneity were available (*P*_h_ <0.1), otherwise the random model (DerSimonian and Laird) was used [11]. Heterogeneity was assessed with I^2^ test, which described the proportion of variation in the log RR that is attributable to genuine differences across studies rather than to random error. I^2^ took values between 0% and 100% with higher values denoting greater degree of heterogeneity (I^2^ = 0% to 25%: no heterogeneity; I^2^ = 25% to 50%: moderate heterogeneity; I^2^ = 50% to 75%: large heterogeneity; I^2^ = 75% to 100%: extreme heterogeneity). Publication bias was assessed using a funnel plot of effect size against standard error.

## Results

### Eligible Studies

**Figure 1:**
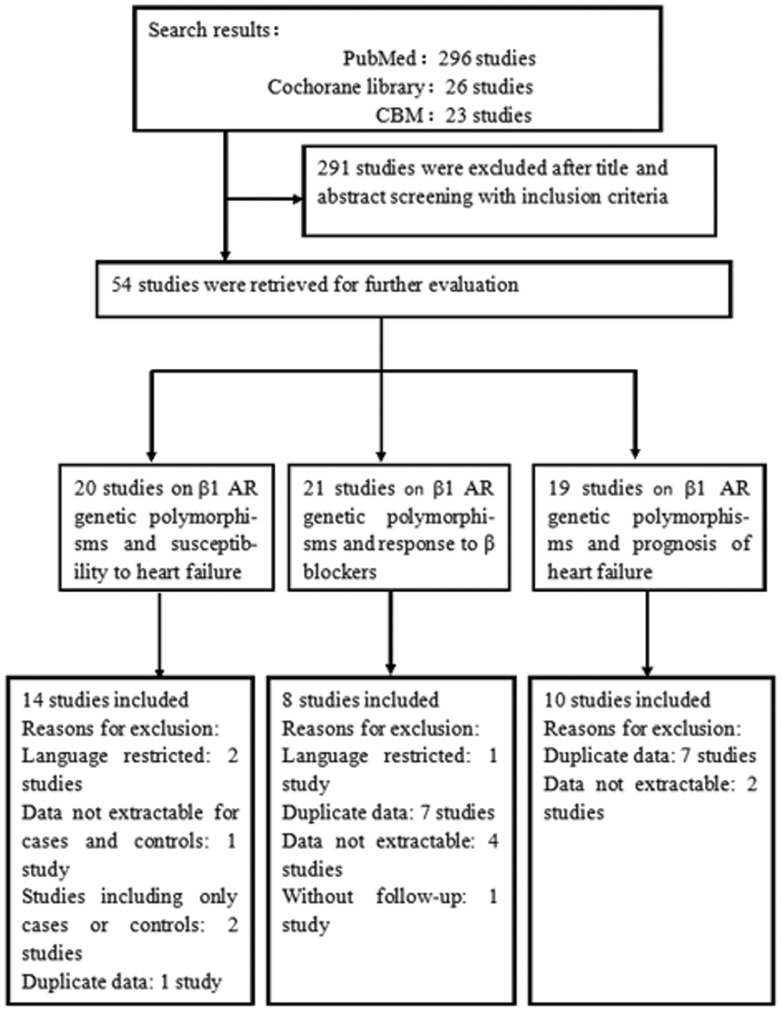
Flow diagram of the study selection process β1-AR: β1 adrenergic receptor.

A total of 345 potentially eligible studies were identified, of which 291 were excluded after reviewing the study abstracts. The retrieved studies were then read in their entirety to assess their appropriateness for inclusion in the meta-analysis. As shown in Table 1, fourteen studies were included for relationship between β1 AR polymorphisms and susceptibility to HF [12]–[25], while 8 studies evaluated the impact of β1 AR polymorphisms on HF therapy response to β-blocker [16], [26]–[32], and 10 studies provided information on association between prognosis and β1 AR polymorphisms [12], [15], [19], [29], [32]–[38]. The reasons why studies were excluded were shown in Figure 1.

**Table 1:** The characters of the 27 eligible studies.

Authors	Year	Region	Ethnicity	Type of study	Sample size(case/control)	Main assessments	Parameter
Wang L et al [12]	2010	China	East Asian	case-control	430/468	Ser49Gly, Arg389Gly	risk of HF, mortality and combined endpoint in2 years
Paczkowska A et al [13]	2009	Poland	White	case-control	97/105	Ser49Gly, Arg389Gly	risk of HF
Woodiwiss AJ et al [14]	2008	South African	Black	case-control	403/429	Arg389Gly	risk of HF
Biolo A et al [15]	2008	Brazil	mixed	case-control	201/141	Ser49Gly, Arg389Gly	risk of HF, mortality in 39.8 months
Yu WP et al [16]	2006	China	East Asian	case-control	105/100	Arg389Gly	risk of HF; ΔHR, ΔLVEF, ΔLVEDd, ΔLVESd with3 months β-blocker treatment
Nonen S et al [17]	2005	Japan	East Asian	case-control	91/119	Ser49Gly, Arg389Gly	risk of HF
Covolo L et al [18]	2004	Italy	White	case-control	256/230	Ser49Gly, Arg389Gly	risk of HF
Magnusson Y et al [19]	2005	Sweden	White	case-control	375/492	Ser49Gly, Arg389Gly	risk of HF, mortality in 5 years
Small KM et al [20]	2002	America	mixed	case-control	159/189	Arg389Gly	risk of HF
Iwai C et al [21]	2002	Japan	East Asian	case-control	163/157	Arg389Gly	risk of HF
Forleo C et al [22]	2007	Italy	White	case-control	189/378	Ser49Gly, Arg389Gly	risk of HF
Tesson F et al [23]	1999	France	White	case-control	426/395	Arg389Gly	risk of HF
Fragoso JM et al [24]	2006	Mexico	Mexican	case-control	47/93	Ser49Gly, Arg389Gly	risk of HF
Podlowski S et al [25]	2000	Germany	White	case-control	37/40	Ser49Gly, Arg389Gly	risk of HF
Metra M et al [26]	2010	Italy	White	prospective	183	Arg389Gly	ΔHR, ΔLVEF, ΔLVEDv, ΔLVESv with 6 monthsβ-blocker treatment
Chen L et al [27]	2007	Australia	White	prospective and retrospective	135	Ser49Gly, Arg389Gly	ΔLVEF, ΔLVEDd, ΔLVESd with 1 year β-blocker treatment
Luo M et al [28]	2007	China	East Asian	prospective	156	Arg389Gly	ΔLVEF, ΔLVESd with 3 months β-blocker treatment
Liggett SB et al [29]	2006	America	Mixed	prospective	515	Arg389Gly	ΔHR, ΔLVEF with β-blocker treatment, mortalityin 12 months
Terra SG et al [29]	2005	America	Mixed	prospective	54	Ser49Gly, Arg389Gly	ΔHR, ΔLVEF, ΔLVEDd, ΔLVESd with 3 monthsβ-blocker treatment
de Groote P et al [31]	2005	France	White	prospective	199	Ser49Gly, Arg389Gly	ΔHR, ΔLVEF with 3 months β-blocker treatment
Mialet Perez J et al [32]	2003	America	NS	retrospective	224	Arg389Gly	ΔLVEF after 6 months β-blocker treatment
Petersen M et al [32]	2011	Denmark	White	retrospective	305	Arg389Gly	mortality in 6.7 years
Leineweber K et al [34]	2010	Germany	White	prospective	226	Ser49Gly, Arg389Gly	mortality in 45 months
Cresci S et al [35]	2009	America	Mixed	prospective	1133	Arg389Gly	mortality and combined endpoint in 6.5 years
White HL et al [36]	2003	UK and Netherland	White	retrospective	600	Arg389Gly	combined endpoint in 12 months
Forleo C et al [37]	2004	Italy	White	prospective	171	Ser49Gly, Arg389Gly	combined endpoint in 33 months
Shin J et al [38]	2007	America	Mixed	prospective	227	Ser49Gly, Arg389Gly	mortality and combined endpoint in 2.8 years

HF, heart failure; LVEF, left ventricular ejection fraction; LVEDd/v, left ventricular end diastolic diameter/volume; LVESd/v, left ventricular end systolic diameter/volume; combined endpoint, including death, heart transplantation and hospitalization; NS, not stated.

### β1 AR Polymorphisms and Susceptibility to HF

A total of 14 case-control studies [12]–[25] containing 2979 patients and 3336 controls provided data on the association between β1 AR polymorphisms and the susceptibility to HF (14 for Arg389Gly and 8 for Ser49Gly, as shown in Table 1). The causes of HF were various, including idiopathic dilated cardiomyopathy (IDCM), hypertension and ischemic cardiomyopathy. Controls were mainly healthy population, and matched for area and ethnicity. Genotypes were determined by polymerase chain reaction-restriction fragment length polymorphism (PCR-RFLP) in all the publications. Studies were conducted in various populations of racial descent: 7 studies for whites, 4 involved East Asians, 1 study for blacks, 1 involved Mexicans, and 1 study was mixed. The distribution of genotypes in the control group deviated from HWE in 2 studies for Ser49Gly [12], [25], and then a sensitivity analysis was carried out excluding these studies to avoid possible genotyping errors and/or population stratification. The distribution of the β1 AR genotypes for cases and controls was shown in Table 2.

**Table 2:** The distribution of the Arg389Gly and Ser49Gly genotype for cases and controls.

		Arg389Arg (or Ser49Ser)		Arg389Gly (or Ser49Gly)		Gly389Gly (or Gly49Gly)		Arg389 (or Ser49)		Gly389 (or Gly49)		
	Polymorphism	HF	control	HF	control	HF	control	HF	control	HF	control	*P* HWE Controls
Wang L 2010	Ser49Gly	270	338	152	130	8	0	692	806	168	130	0.00048
Wang L 2010	Arg389Gly	263	316	135	139	32	13	661	771	199	165	0.62323
Paczkowska A 2009	Ser49Gly	76	87	21	17	0	1	173	191	21	19	0.86769
Paczkowska A 2009	Arg389Gly	57	50	35	47	5	8	149	147	45	63	0.50042
Woodiwiss AJ 2008	Arg389Gly	200	210	161	172	42	47	561	592	245	266	0.19305
Biolo A 2008	Ser49Gly	146	105	50	30	5	6	342	240	60	42	0.05634
Biolo A 2008	Arg389Gly	112	81	72	52	17	8	296	214	106	68	0.92718
Yu WP 2006	Arg389Gly	54	53	42	40	9	7	150	146	60	54	0.88303
Nonen S 2005	Ser49Gly	66	83	21	33	4	3	153	199	29	39	0.896
Nonen S 2005	Arg389Gly	60	79	26	35	5	5	146	193	36	45	0.65567
Covolo L 2004	Arg389Gly	119	122	116	90	21	18	354	334	158	126	0.8053
Magnusson Y 2005	Ser49Gly	255	333	110	148	10	11	620	814	130	170	0.24506
Magnusson Y 2005	Arg389Gly	218	266	140	201	15	25	576	733	170	251	0.09614
Small KM 2002 (blacks)	Arg389Gly	43	63	34	34	4	8	120	160	42	50	0.27066
Small KM 2002 (whites)	Arg389Gly	23	23	36	48	19	13	82	94	74	74	0.14432
Forleo C 2007	Ser49Gly	137	318	51	58	1	2	325	694	53	62	0.711
Forleo C 2007	Arg389Gly	100	173	78	159	11	46	278	505	100	251	0.31498
Tesson F 1999	Arg389Gly	261	231	140	136	25	28	662	598	190	192	0.20168
Fragoso JM 2006	Ser49Gly	25	61	19	28	3	4	69	150	25	36	0.73168
Fragoso JM 2006	Arg389Gly	24	69	20	20	3	4	68	158	26	28	0.12488
Iwai C 2002	Arg389Gly	88	74	54	71	21	12	230	219	96	95	0.36985
Podlowski S 2000	Ser49Gly	31	40	5	0	1	0	67	80	7	0	–
Podlowski S 2000	Arg389Gly	19	21	16	18	2	1	54	60	20	20	0.2059

HWE: Hardy–Weinberg Equilibrium.

The results for the associations between the β1 AR polymorphisms and the risk of HF were shown in Table 3. In general population, the Arg389Gly polymorphism was not significantly associated with HF for all genetic models, the heterogeneity among studies was significant (Gly vs. Arg: *P*_h_ = 0.01, I^2^ = 52%). In subgroup analysis by ethnicity, in East Asians Gly389 allele increased the susceptibility to HF (RR = 1.10; 95% CI: 1.01–1.19, *P* = 0.03), and Gly389 homozygote was significantly associated with a 35% increased risk of HF compared with Arg389 carrier (RR = 1.35; 95% CI: 1.16–1.57, *P*<0.01); in contrast, Gly389 allele (RR = 0.94; 95% CI: 0.89–1.00, *P* = 0.06) and Gly389 homozygote (RR = 0.84; 95% CI: 0.71–1.00, *P* = 0.05) trend to decrease the risk of HF in whites. Among blacks, there was not a significant relationship between the Arg389Gly polymorphism and HF.

**Table 3:** RRs and Heterogeneity Results for the Genetic Contrasts of Arg389Gly and Ser49Gly β1 AR Polymorphisms for HF.

Polymorphism	Ethnicity	RR (95% CI)	Studies	I^2^, %	*P*_h_	Overall effect, *P*
Arg389Gly						
Gly vs. Arg	All	1.01 (0.95, 1.08)	14	52	0.01	0.66
	Asian	1.10 (1.01, 1.19)	4	26	0.25	0.03
	White	0.94 (0.89, 1.00)	7	38	0.14	0.06
	Black	1.00 (0.91, 1.10)	2	0	0.5	0.98
Gly389 carrier vs. Arg389Arg	All	1.00 (0.93, 1.08)	14	41	0.05	0.98
	Asian	1.05 (0.95, 1.17)	4	35	0.2	0.31
	White	0.95 (0.88, 1.03)	7	34	0.17	0.19
	Black	0.98 (0.86, 1.12)	2	0	0.84	0.77
Gly389Gly vs. Arg389 carrier	All	1.08 (0.93, 1.24)	14	50	0.01	0.31
	Asian	1.35 (1.16, 1.57)	4	0	0.48	0.0001
	White	0.84 (0.71, 1.00)	7	0	0.46	0.05
	Black	1.05 (0.87, 1.27)	2	50	0.16	0.62
Ser49Gly						
Gly vs. Ser	All	1.22 (1.04, 1.43)	8	80	<0.01	0.02
	Sensitivity	1.11 (0.97, 1.27)	6	51	0.07	0.13
	Asian	1.18 (1.06, 1.31)	2	44	0.18	0.003
	White	1.34 (0.95, 1.90)	4	90	<0.01	0.1
Gly49 carrier vs. Ser49Ser	All	1.33 (1.03, 1.72)	8	52	0.04	0.03
	Sensitivity	1.25 (0.94, 1.66)	6	48	0.08	0.12
	Asian	1.24 (0.72, 2.13)	2	64	0.09	0.44
	White	1.48 (0.86, 2.57)	4	72	0.01	0.16
Gly49Gly vs. Ser49 carrier	All	1.24 (0.74, 2.08)	8	81	<0.01	0.42
	Sensitivity	1.03 (0.76, 1.39)	6	0	0.84	0.86
	Asian	1.72 (0.95, 3.12)	2	69	0.07	0.07
	White	1.08 (0.73, 1.62)	4	0	0.76	0.69

For the Ser49Gly polymorphism, overall, the heterogeneity among studies was significant (*P*_h_<0.01, I^2^ = 80%). The Gly49 allele significantly increased HF risk (RR = 1.22; 95% CI: 1.04–1.43, *P* = 0.02) compared with Ser49, while Gly49 carrier had significantly higher risk of HF than Ser49Ser homozygote (RR = 1.33; 95% CI: 1.03–1.72, *P* = 0.03). But neither of the associations was significant in sensitivity analysis. In ethnicity subgroup analysis, Gly49 significantly increased HF risk compared with Ser49 (RR = 1.18; 95% CI: 1.06–1.31, *P*<0.01) in East Asians, however, it was not robust either. No association between the risk of HF and Ser49Gly polymorphism was found in whites.

### β1 AR Polymorphisms and Response to β-blocker Therapy

Eight studies [16], [26]–[32] evaluated the impact of β1 AR polymorphisms on response to β-blocker therapy: 2 involved East Asians, 3 studies for whites, and 3 studies were mixed (Americans, mainly composed of blacks and Caucasians). ΔHR and parameters of LV remodeling (ΔLVEF, ΔLVEDd/v, and ΔLVESd/v) were used to assess the response to β-blocker therapy with at least 3 months follow up. All the 1561 patients with a LVEF ≤45% received concomitant drug therapy, which included β-blocker, angiotensin converting-enzyme (ACE) inhibitor (or an angiotensin receptor blocker if the ACE inhibitor was not tolerated), spironolactone, digoxin, duretics and so on. The kinds of β-blockers were various, comprised selective β1-blockers (metoprolol, bisoprolol) and non-selective β-blockers (carvidilol, bucindolol). And the dose of β-blockers was the target dose according to guideline or a maximum tolerated dose. The LVEF, LVEDd/v and LVESd/v were measured with echocardiogram or radionuclide ventriculography.

**Figure 2:**
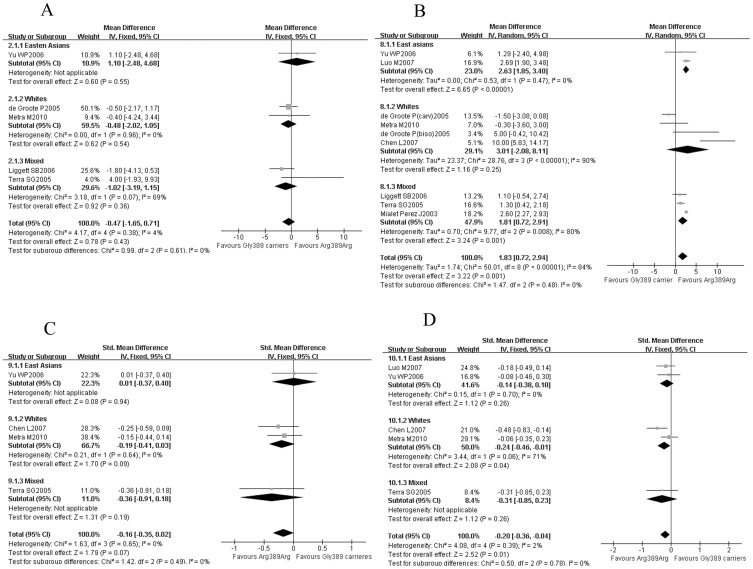
Arg389 homozygotes vs. Gly389 carriers in different ethnics: the response to β-blockers. (A) the reduction of HR; (B) the improvements of LVEF; (C) the improvements of LVEDd/v; (D) the improvements of LVESd/v. Gly389 carriers: including Arg389Gly and Gly389 homozygotes; CI: confidence interval; HR: heart rate; LVEF: left ventricular ejection fraction; LVEDd/v: left ventricular end-diastolic diameter/volume; LVESd/v: left ventricular end-systolic diameter/volume.

Five studies [16], [26], [29]–[31] including 1056 patients provide information on the reduction of heart rate after β-blocker therapy. There was not a significant difference in the reduction of heart rate between Arg389 homozygotes and Gly389 carriers (WMD = −0.47, 95% CI: −1.65–0.71, *P* = 0.43, Figure 2A). Even in different ethnics, the HR reductions with β-blockers treatment were comparable in the Arg389 homozygotes and Gly389 carriers.

Compared with Gly389 carriers, overall, there was a significant improvement in LV remodeling in Arg389 homozygotes. Eight studies [16], [26]–[32] containing 1602 patients indicated that the improvement of LVEF was better in Arg389 homozygotes (WMD = 1.83, 95% CI: 0.72–2.94, *P*<0.01, Figure 2B). And another meta-analysis including 4 studies [16], [26], [27], [29] and 477 patients showed that the LVEDd/v improvement of Arg389 homozygotes trended to be better than Gly389 carriers (SMD = −0.16, 95% CI = −0.35–0.02, *P* = 0.07, Figure 2C). And it was also found that the LVESd/v improvement of Arg389 homozygotes was significantly greater than Gly389 carriers (SMD = −0.20, 95% CI: −0.36– −0.04, *P* = 0.01, Figure 2D) from a meta-analysis of 5 studies [16], [26], [27], [28], [29] and 633 patients.

In further subgroup analysis, it was found that the Arg389 homozygotes were associated with a better LVEF improvement in East Asians (WMD = 2.63, 95% CI: 1.85–3.40, P<0.01, Figure 2B) and mixed population (WMD = 1.81, 95% CI: 0.72–2.91, P<0.01, Figure 2B); while among white patients, the Arg389 homozygotes made a better improvement of LVESd/v (SMD = −0.24, 95% CI = −0.46– −0.01, P = 0.04, Figure 2D) and also a trend of better improvement of LVEDd/v (SMD = −0.19, 95% CI = −0.41–0.03, P = 0.09, Figure 2C ).

**Figure 3:**
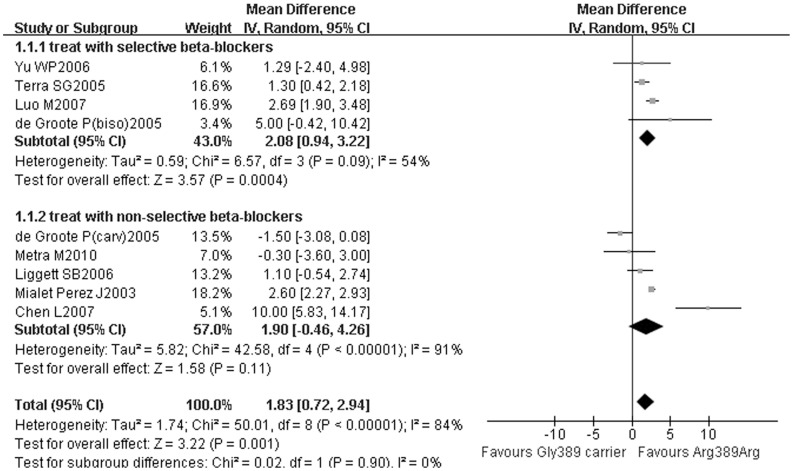
Arg389 homozygotes vs. Gly389 carriers with selective or non-selective β-blockers: the improvements of LVEF. Gly389 carrier: including Arg389Gly and Gly389 homozygotes; CI: confidence interval; LVEF: left ventricular ejection fraction.

In another subgroup analysis, the LVEF improvement of Arg389 homozygotes was significantly greater than Gly389 carriers (SMD = 2.08, 95% CI: 0.94–3.22, *P*<0.01, Figure 3) in patients treated with selective β1-blockers, but non-selective β-blockers did not achieve different LVEF improvements between Arg389 homozygotes and Gly389 carriers (SMD = 1.90, 95% CI: −0.46–4.26, *P* = 0.11, Figure 3).

### β1 AR Polymorphisms and Prognosis of HF

A total of 10 studies evaluated the impact of β1 AR polymorphisms on HF mortality and/or combined endpoint incidence. In 2 of them, the kind of β-blocker was fixed (BEST research: bucindolol, MERIT-HF: metoprolol CR/XL). Patients in the other 8 studies were mostly treated with β-blockers (at least 70%), and the kinds and dose of β-blockers (metoprolol, bisoprolol, carvidilol and other β-blocker) were decided by the subjects’ physicians. The patients were mostly whites in the 10 studies, in which the causes of HF were various (ischemic/IDCM/hypertension).

**Figure 4:**
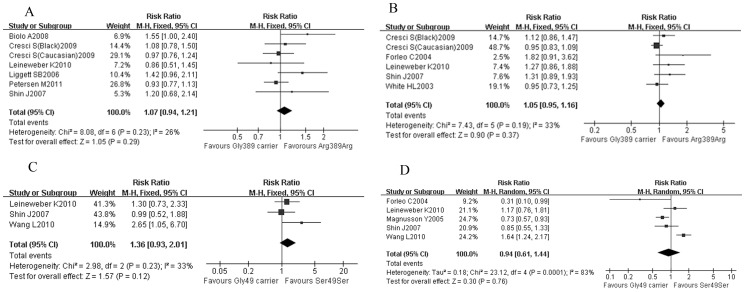
Impact of β1-AR polymorphisms on prognosis of HF. (A) Arg389 homozygotes vs. Gly389 carriers: all-cause mortality; (B) Arg389 homozygotes vs. Gly389 carriers: combined end-points; (C) Ser49 homozygotes vs. Gly49 carriers: all-cause mortality; (D) Ser49 homozygotes vs. Gly49 carriers: combined end-points. Gly389 carrier: including Arg389Gly and Gly389 homozygotes; Gly49 carrier: including Ser49Gly and Gly49 homozygotes; CI: confidence interval; combined end-points including death, heart transplantation and hospitalization.

There was no significant difference between Arg389 homozygotes and Gly389 carriers in all-cause mortality (RR = 1.07; 95% CI: 0.94–1.21, *P* = 0.29, Figure 4A) or combined end-point (RR = 1.05; 95% CI: 0.95–1.16, *P* = 0.37, Figure 4B). And all-cause mortality of Ser49 homozygotes was not significantly different with Gly49 carriers (RR = 1.36; 95% CI: 0.93–2.01, *P* = 0.12, Figure 4C), so it was with the combined end-point (RR = 0.94; 95% CI: 0.61–1.44, *P* = 0.76, Figure 4D).

### Sensitivity Analysis

The small sample studies (sample size <100) [25], [29] involved in our meta-analysis were deleted to reflect the influence of them on our data and conclusion. And the pooled RRs were not significantly altered, indicating that the results were robust.

### Publication Bias

Begg’s funnel plots were performed to access the publication bias of susceptibility to HF, response to β-blocker therapy and prognosis of HF. The funnel plots appeared symmetric, suggesting the absence of publication bias (data not shown).

## Discussion

The purpose of diagnosing and treating HF is bringing about a reduction of mortality and morbidity. Preventive measurements can be undertaken early according to the risk factor for the development of HF, while appropriate therapeutic decision would be made by accurate prediction of response to β-blockers. Therefore, predictors of susceptibility to HF and response to β-blockers treatment are needed in clinical practice. Sustained sympathetic system activation has been shown to be deleterious to the failing heart, and the transmitters of this system, adrenaline and noradrenaline act on β-receptors situated on cardiomyocytes, which are mostly of the β1-subtype [8]. The β-blockers recommended by the guidelines – metoprolol, bisoprolol, carvidilol can block β1- receptors as their common function [1], [2]. Therefore, the β1 adrenergic receptor plays decisive role in the development and treatment of HF. In vitro and in vivo experiments showed that agonist-related downregulation in Gly49 variant was significantly greater than Ser49 [39]. It was also found that Arg389 receptor would have a greater agonist-promoted coupling to Gs/adenylyl cyclase compared with Gly389 [40], and β1 AR Arg389 desensitized more rapidly than the Gly389 variant [41]. Since the important role of β1 AR in HF, it was inferred that the two functional polymorphisms would predict the susceptibility to HF, the response to β-blockers treatment, and even the prognosis of HF. However, there was no large scale study to verify the relationship.

We have performed a meta-analysis on the impact of β1 adrenergic receptor polymorphisms on susceptibility to heart failure, response to β-blocker therapy and HF prognosis, including data on over 7000 patients and 3000 healthy controls. It has been found that Gly389 allele and Gly389 homozygote increased the risk of HF in East Asians, but trended to decrease the risk of HF in whites. Overall the response to β-blockers in Arg389 homozygote was greater than that in Gly389 carrier while the reductions of HR were similar. But the Arg389 homozygotes did not confer a significant prognosis benefit in heart failure patients. The Ser49Gly polymorphism was associated with neither risk nor prognosis of HF.

There was evidence in transgenic mice to show that Arg389 allele was a risk factor of HF [42], while there may be a role for the Ser49Gly polymorphism in the susceptibility to HF [43]. In the present meta-analysis, Gly49 allele increased the HF risk in the general population and in East Asians, but the result was not robust in sensitivity analysis. In the studies with the controls not in HWE, the lack of HWE indicates genotyping errors, population stratification, and selection bias, which could be potential sources of biases [9]. Recently, a meta-analysis [44] found that the Gly389Gly significantly increased risk of idiopathic dilated cardiomyopathy (IDCM) in Asians, which consisted with our result. But in that study, Arg389Gly was not associated with susceptibility to IDCM in Europeans. In our study, the causes of HF included IDCM, hypertension and ischemic cardiomyopathy. The important role of Arg389Gly in essential hypertension [45] and LV remodeling in patients following acute myocardial infarction (AMI) [46] could explain that Arg389Gly polymorphism affected the risk of HF but did not associate with susceptibility to IDCM in whites.

Furthermore, as a complex pathophysiological process, heart failure was involved in multi-genetic effection, thus other functional genes that affected the susceptibility to HF may mask the influence of β1 adrenergic receptor polymorphisms in different ethnics. In addition, environmental interactions (e.g., smoking, physical activity, and diet) may also play a role in the pathogenesis of HF [47]. In the future, the polymorphisms within haplotypes combined with environmental interactions can be a risk stratification tool of HF rather than the individual polymorphism.

Experiments in mice and healthy volunteers showed that the reduction of HR to β-blocker in Arg389 homozygotes was greater than that in Gly389 carriers [43], [48]. However, in our meta-analysis the reductions of HR were comparable, which might be related with the downregulation of myocardial β1 AR in HF patients. While the improvements of LVEF and LVESd/v were significantly better in Arg389 homozygotes, which also performed a trend for greater reduction of LVEDd/v. In clinic practice, doctors treat the patient would titrate β-blockers to achieve appropriate heart rate reduction. According to the results, Arg389Gly polymorphism may be a predictor of response to β-blocker treatment in HF patients, which is independent of HR. These findings would be useful in making individualized therapeutic decision in the future.

In another subgroup analysis, the Arg389 homozygotes performed a greater LVEF improvement to selective β1-blockers therapy than Gly389 carriers, but no differences were conferred by genotypes in the patients treated with non-selective β-blockers, as non-selective β-blockers also block β2 and α1 AR. The LVEF is an important indicator of response to treatment. Blockade of the β1AR is certainly responsible for a large degree of the improvement seen in HF, such as LVEF improvement and survival benefits, but it is not the sole mechanism. Previous studies have suggested that the release of norepinephrine is partly regulated by prejunctional β2-adrenergic receptors [49], and that α1-ARs can also increase contractility equal to β -ARs in failing hearts [50]. As the importance of β2 and α1 AR in failing hearts, the role of β1 AR in patients who were treated with non-selective beta-blockers was weakened. Thus the association between the improvement of LVEF and Arg389Gly polymorphism was not significant among patients treated with non-selective β-blockers.

As the progression of heart failure is associated with LVEF, it is expected the Arg389 homozygotes would get a better efficacy by selective β1-blockers therapy and Gly389 carriers might benefit more from non-selective β-blockers, which needs further investigation.

In a study of 54 patients with HF, individuals with the Gly49 variant had greater improvement in LVEDd [29]. However, no association between Ser49Gly polymorphism and the change of LVEF was found [27], [31]. The being few studies, these findings need to be further confirmed.

Although the therapeutic response to β-blockers was influenced by the β1 AR polymorphisms, no differences were found in the prognosis of heart failure. In theory, a better beta-blocker response might lead to potential adverse outcomes, such as HF deterioration, hypotension and bradycardia. But by now, there has been no data supporting the association between the β1 genetic polymorphisms and the adverse outcomes in prognosis. It is also believed that in the failing heart the density of β AR, especially the β1 AR, kept decreasing [51]. In our meta-analysis, the follow-up time of studies on prognosis of HF was relatively longer than that of studies on β-blocker responses. Thus the density of β1-AR in studies on prognosis of HF was more decreased than that in studies on beta-blocker responses. Therefore, the β1 AR played a more important role in response to β-blockers than that in prognosis of HF. Recently, a meta-analysis [52] has shown that the patients enrolled in the United States were associated with a lower magnitude of survival benefit to β-blockade than the ones from the rest of the world. The result demonstrated that the determining prognosis in HF is complex. Besides genetic factors, the cultural or social differences in disease management may also cover the impact of β1-AR polymorphisms. In addition, the sample size would have been too small to detect the differences.

In the future, large sample clinical trials which investigate both the response to beta-blockers and prognosis in the same group of patients may make more sense for this problem.

### Limitation

First of all, we evaluated the relationships between heart failure and the two functional β1 AR polymorphisms, Ser49Gly and Arg389Gly respectively. Since the two polymorphisms are in strong linkage disequilibrium (LD) [53], the combined effects in haplotypes may be more decisive in susceptibility to HF, response to β-blocker therapy, and prognosis.

Secondly, in the 10 studies for prognosis of HF, the percentage of patients treated with beta-blockers ranged from 70% to 100%. The non-treated cases could not be excluded so that our results would inevitably be compromised. It’s a limitation of our meta-analysis. But in reality, some patients with heart failure due to the presence of contraindications cannot use β-blockers. Therefore, we believe that the current results were closer to clinical practice.

At last, the sample size for each study was relatively small, even though all the studies in which the data could be achieved were collected for analysis, yet prospective studies with large sample size are warranted.

### Conclusion

Based on the present meta-analysis, we found that the Gly389 allele and Gly389 homozygote were associated with the increasing risk of HF in East Asians; but in whites, Gly389 allele and Gly389 homozygote trended to decrease the risk of HF. The Ser49Gly polymorphism of β1 receptor was not associated with risk of HF. With the similar reduction of heart rate, overall, the Arg389 homozygotes performed a better response to β-blocker therapy. Furthermore, the Arg389 homozygotes were significantly associated with better LVEF improvement in East Asians and mixed population. And in white people, the Arg389 homozygotes made a greater LVESd/v improvement and trended to be associated with better LVEDd/v improvement. However, neither of the two β1 AR polymorphisms impacted the prognosis of HF based on our data. The results of our meta-analysis would provide a reference for individualized treatment of HF, and are also helpful in future prospective clinical trials.

## Supporting Information

Checklist S1 (DOC)
